# Use of Contraception and Attitudes towards Contraceptive Use in Swedish Women - A Nationwide Survey

**DOI:** 10.1371/journal.pone.0125990

**Published:** 2015-05-20

**Authors:** Helena Kopp Kallner, Louise Thunell, Jan Brynhildsen, Mia Lindeberg, Kristina Gemzell Danielsson

**Affiliations:** 1 Department of Women´s and Children´s Health, Karolinska Institutet, Stockholm, Sweden; 2 Department of Clinical Sciences at Danderyd Hospital, Karolinska Institutet, Stockholm, Sweden; 3 Department of Obstetrics and Gynecology, Skåne University Hospital, Malmö, Sweden; 4 Division of Obstetrics and Gynecology, Department of Clinical and Experimental Medicine, Faculty of Health Sciences, Linköping University, Linköping, Sweden; 5 MSD, Stockholm, Sweden; NHS lothian and University of Edinburgh, UNITED KINGDOM

## Abstract

**Objective:**

To describe contraceptive use and attitudes towards contraceptive use in Sweden which has the highest abortion rate in Western Europe. Secondary objectives were to investigate knowledge of contraceptive methods and outcomes of unplanned and unwanted pregnancies.

**Design:**

Telephone survey.

**Setting:**

National survey of women living in Sweden.

**Population:**

Women between 16 and 49 years.

**Methods:**

The survey contained 22 questions with free text and multi choice answers on demographics, contraceptive use, knowledge of and attitudes towards contraception, the importance of monthly bleeding and experience of unintended pregnancy.

**Main Outcome Measures:**

Distribution of use of contraceptive methods and non-use of contraception among Swedish women. Prevalence and outcome of unintended pregnancies.

**Results:**

A total of 1001 women participated in the survey. Of all women, 721/1001 (72.1%) currently used contraception whereas 268/1001 (26.8%) women did not. Long acting reversible contraception, (LARC; implant and intra uterine contraception) was used by 24.3% of women. The unmet need of contraception in Sweden was estimated at 8.9% (89/1001 women). A total of 781 (78%) women had never experienced an unintended pregnancy whereas 220 (22%) women had had at least one unintended pregnancy. Users and non-users alike stated that one of the most important characteristics of a contraceptive method is its effectiveness.

**Conclusions:**

Sweden has a large unmet need for contraception. Furthermore, a large proportion of women have experienced at least one unintended pregnancy. Increasing contraceptive use and promotion of LARC is a possible way forward in the effort to reduce the rates of unwanted pregnancies.

## Introduction

Access to contraceptive methods and legal access to abortion is linked to low rates of unintended pregnancy. However, Sweden has a unique position in Europe as having the highest rate of pregnancy termination in western Europe[1] in spite of easy access to contraceptive counseling and effective contraception being available. The Swedish model includes nurse midwives prescribing most contraceptives in Sweden. All nurse midwives are licensed to prescribe contraceptives to healthy women. The only physicians prescribing contraceptives in Sweden are gynecologists. There is no contraceptive prescription by general practitioners. In Sweden there are maternity and youth clinics where contraception and contraceptive counseling is readily available and in almost all of Sweden certain contraception is at subsidized cost or even for free for youth. Subsidies differ in available methods and amounts women have to pay depending on the local authorities. The reason for creating the Swedish model was a belief that ready access and subsidies would increase contraceptive use and lower the numbers of unwanted pregnancy. Unfortunately during the past decades unwanted pregnancies have stayed at a constant high level with the number of abortions per 1000 women hovering at around 20. Sweden also differs from the other Nordic countries which have significantly lower rates of abortion.

To be able to understand why Sweden has a high number of unwanted pregnancies in spite of the large number of contraceptive providers and subsidies, knowledge of attitudes towards contraception and the contraceptive habits of Swedish women is needed. This knowledge can lead to appropriate action. Official statistics only cover use of prescribed hormonal contraception. However, contraceptive use may be overestimated using the register due to adherence issues. The prevalence of use of methods not requiring prescriptions is unknown.

Effectiveness of contraceptive methods is judged to be important when women decide what method to use [2]. The “typical use” effectiveness rather than the “perfect use” effectiveness of contraceptive methods should be considered in contraceptive counseling[3]. However, the typical use effectiveness of contraceptive methods may not be common knowledge among providers or women. It has been shown that younger women have less user adherence to methods that require a daily intake[4]. However, in Sweden the national guidelines for contraceptive use recommended that combined hormonal contraceptives should be the first method of choice for young women. The implant and intrauterine contraception (IUCs; intrauterine hormonal systems, copper intra uterine devices) should be used as a second choice. These recommendations were changed in spring 2014.[5]

Recently, the importance of long acting reversible contraceptive methods (LARC; implants and IUCs) in reducing the rate of unwanted pregnancy, abortion and repeat abortion has been shown.[4, 6, 7] Knowledge of the distribution of LARC, short acting reversible contraception (SARC; contraceptive pills, the combined hormonal contraceptive patch and ring, and progestin only injectables), permanent methods and other methods could give us more information on how to promote LARC use among the age groups most susceptible to unwanted pregnancies. Structured counseling and the cost of contraception can change the contraceptive method that women decide to use.[4, 8]

In order to obtain the information required for appropriate action against the high numbers of unwanted pregnancies, a nationwide survey of 1000 women representative of the Swedish female population was conducted. The primary object of the survey was to describe contraceptive use and attitudes towards contraceptive use in Sweden. Secondary objectives were to investigate the knowledge women have of contraceptive methods, why women choose not to use contraception and how they handle unplanned and unwanted pregnancies.

## Material and Methods

The telephone survey was performed by TNS Sifo´s specialists in healthcare surveys—Navigare. TNS Sifo is the Swedish branch of TNS- a global company specialized in market surveys. It was estimated that 1000 women would be sufficient to be able to conduct valid subgroup analyses of different age intervals. Women between the ages 16–49 were included and identified through the database “PAR KONSUMENT” which covers 90% of all individuals above the age of 16 years in Sweden. A separate sample was drawn for women aged 16–20 in order to get a good stratification for young women. More information on the database in available in supplementary information (S1 Document). To correct for the fact that younger women are less represented in telephone registers the proportion of young individuals is multiplied by a factor of 1.2 according to standard operating procedure. Both fixed and mobile telephone numbers were used to contact the women. Up to 3 attempts were made to reach each individual woman, using both fixed and mobile numbers, before they were included in the contact failure group. Each individual in the contact failure group was replaced by a woman in the same age category.

The distribution of the demographical characteristics of individuals in the offered sample is always compared to the expected distribution supplied by the Tax Authority. The demographical characteristics that are compared are the ones present in the Tax Authority register such as age and areas where people live. Therefore, these are the characteristics that can be corrected for. The Tax Authority does not offer statistics on current income or ethnic background to commercial companies when matched to personal identification numbers. Therefore, it is impossible to correct for these characteristics. Thus, the bias which remains cannot be corrected for as long as the study is performed as a telephone survey.

All women who were reached were informed about the objectives of the survey prior to questioning and that all personal information would be coded and maintained confidential and would not be available for the commissioner of the report. All women were informed that the survey was a collaboration between a pharmaceutical company (MSD Sweden) and independent researchers. Immigrant women were excluded if they had insufficient knowledge of the Swedish language to understand the questions which was judged by the professional interviewer.

### Ethics statement

All consent was obtained verbally as is customary in telephone surveys. All women had a choice to answer questions and to quit the telephone survey at any time. Written consent was therefore not considered as a necessary part of the protocol. The verbal informed consent was recorded in the subject file. Parental consent was obtained by the interviewer in case the interviewed person was a minor. The parental consent was recorded in the subject file. Verbal subject and parental consent and the study as such were approved by the Ethical Review Board of Karolinska Institutet, Stockholm, Sweden (permit nr 2013/1212-31/5).

The survey contained 10 questions on demographic characteristics of women such as age, which part of the country and what kind of community they live in (city, country side large city etc), employment status, educational level, civil status, income in the household, and ethnic background (born in Sweden, parents born in Sweden etc).

There were 22 questions on choice of contraceptive method, knowledge on contraceptive methods, criteria on aspects important for contraceptive choice, reasons for not using contraception (for women not currently using contraception), perceived knowledge on the efficacy of different contraceptive methods, opinion on importance of monthly bleeding or absence thereof, perceived problems during contraceptive use, opinions about how information on contraception can be improved, and experience of unwanted pregnancy Answers were open and of multi-choice character. The surveys structure and questions were modeled from an Austrian contraceptive survey performed in 2012.[9] It took approximately 15 minutes to complete. The survey was piloted orally in 5 women and after minor adjustments to a few questions the face validity was considered good.

## Results

**Fig 1:**
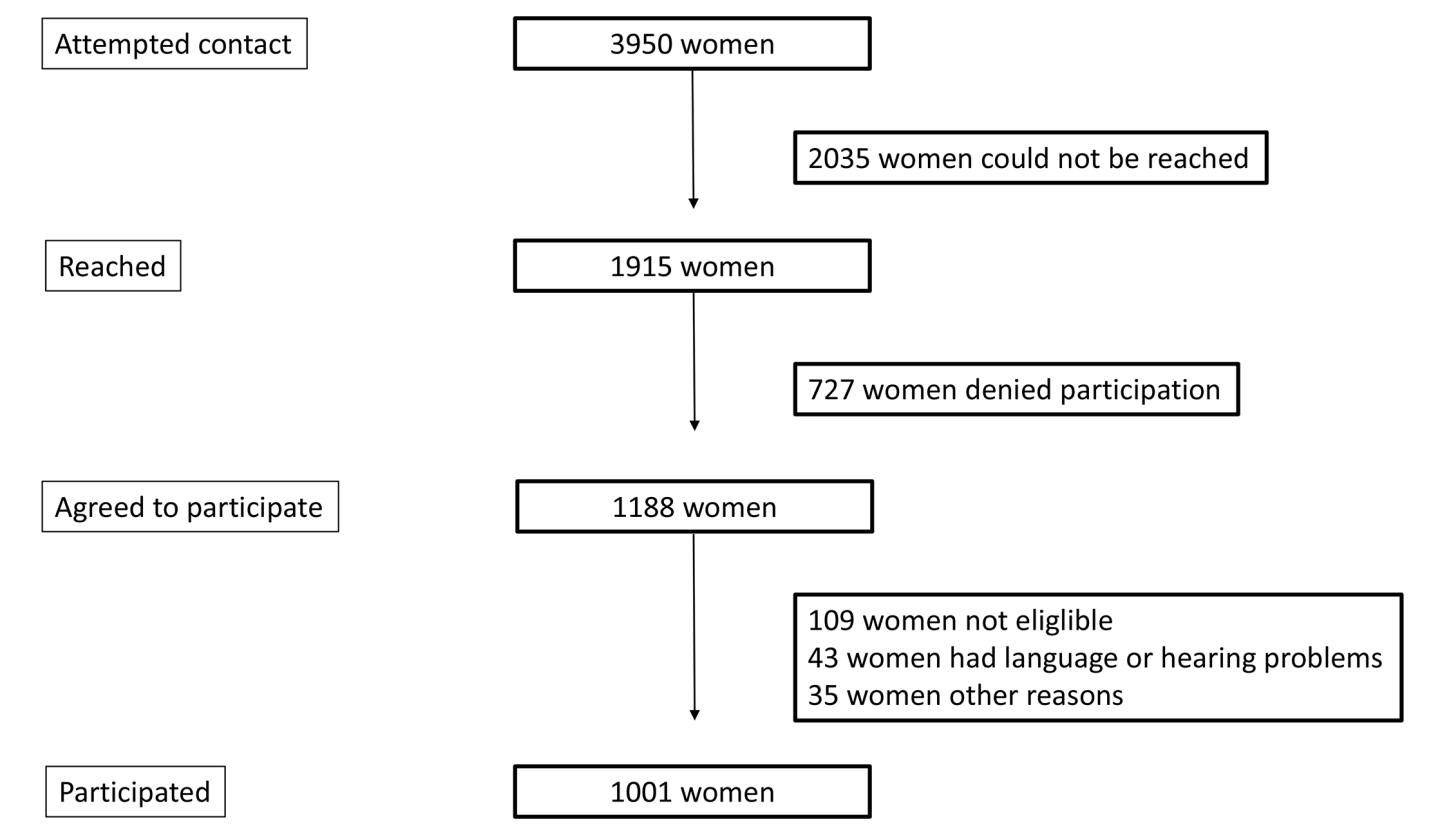
Flow chart.

In total 3950 women between 16–49 years women were contacted. The flow of patients is described in Fig 1. A total of 2035 women which corresponds to 51.5% of all women could not be reached within three attempts. A number of women (727 women, 18.4%) actively stated that they did not want to take part in the survey. The reasons for declining were not having enough time, did not feel like participating or having a principle of not participating in surveys. Women who could not be reached or who declined to participate were replaced from within their age demographic group until 1000 women had responded (The complete dataset is available online as supplementary information, S1 Database). The known demographic information of the women who declined participation was analyzed and found not to differ from that of the general Swedish female population (calculated from known means for the general population, information obtained from the National Tax Authority in Sweden, analyzed with 95% confidence intervals, results not shown). There were slightly fewer immigrant women who participated in the survey (8%) compared to the population of this age groups (20%)[10]. However, in other aspects the women were found to represent the Swedish female population well with reference to where they lived, civil status, employment status, age, income, and level of education according to census information in Sweden (results not shown). Of all women, 721/1001 (72.1%) women currently used contraception, 268/1001 women (26.8%) did not use contraception and 12/1001 (1.2%) had stopped using contraception sometime the last 12 months.

**Fig 2:**
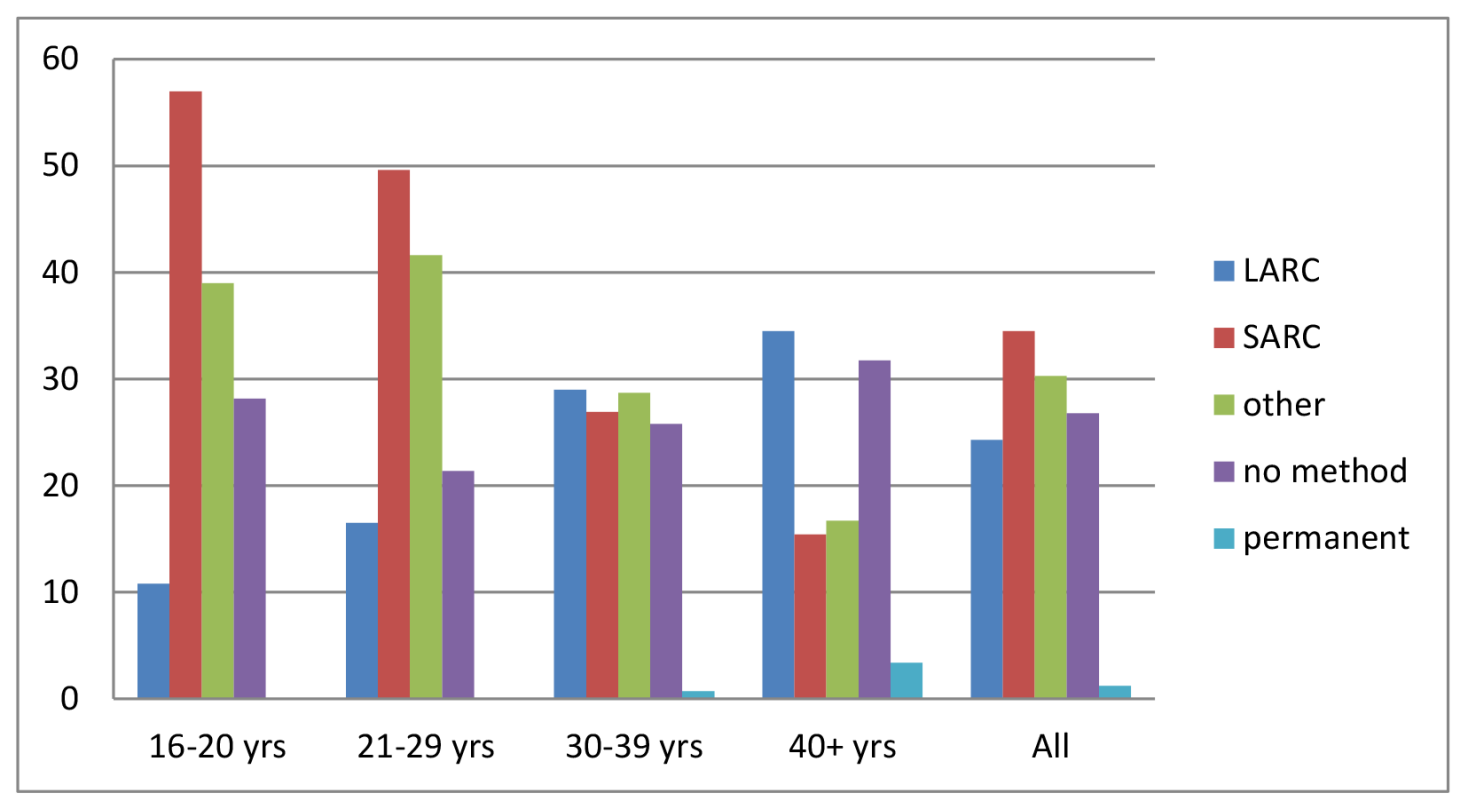
Percentage of women using a contraceptive method depending on age group.

The distribution of contraceptive methods used by all women and in different age groups is presented in Fig 2. Use of SARC was highest in the younger women and thereafter became lower with increasing age. LARC use was lowest in younger age groups and thereafter rose with increasing age. The use of other methods such as the condom or emergency contraceptive pills had a peak at 20–29 years and thereafter fell.

Of all users of contraception, 11% (79/721) stated that they would use another method if all methods cost the same. The contraceptive implant was the most desired contraceptive method among these women.

The reasons for not using contraception differed in different age groups. Reasons for not using contraception in different age groups are given in Table 1. Awareness of the progestin only pill, intra uterine hormonal systems, copper intra uterine devices and the implant was significantly higher in women currently using contraception (Table 2). Awareness of other contraceptive methods did not depend on the women being a current user of contraception. The highest awareness was of the combined hormonal contraceptive pill of which 95–96% of women were aware. The group of women not using contraception had a lower proportion of women stating spontaneous awareness of all methods except for the male condom and coitus interruptus.

**Table 1:** Reasons for not using contraception.

	Age group			
Reason for not using contraception	16–20 (n = 47)	21–29 (n = 59)	30–39 (n = 72)	40–49 (n = 64)
Not sexually active	31	19	7	17
Desire to have baby/gave birth during the period	1	19	38	7
Woman or partner Infertile/sterilized	0	2	65	33
Do not want to use contraception/fear of hormones	0	7	7	14
No need (unspecified)	3	1	6	7
Sex seldomly	0	5	4	3
Side effects	5	3	3	3
Entered menopaus	0	0	0	8
Same sex partners	0	3	3	0
Laziness	3	1	1	0
No method works/have not found good method	0	1	3	1
Other	3	3	4	4
Do not know/no answer	2	1	0	4
Total	48	65	141	101

Women could state more than one reason for not using contraception.

**Table 2:** Awareness of different contraceptive methods.

Contraceptive method	Current user n = 721(%)	Current non usern = 280(%)	p-value
Combined oral contraception	692 (96)	266 (95)	0,49
Copper intrauterine device	495 (67)	154 (58)	0,007*
Male condom	447 (62)	185 (66)	0,24
Intra uterine hormonal system	470 (64)	142 (54)	0,003*
Implant	444 (60)	142 (54)	0,05*
Progestin only pill	237 (32)	63 (24)	0,01*
Progestin only injection	224 (31)	70 (25)	0,06
Diaphragm	202 (28)	70 (25)	0,34
Combined hormonal contraceptive vaginal ring	93 (13)	22 (8)	0,057
Combined hormonal contraceptive patch	80 (11)	25 (9)	0,36
Sterilization	22 (3)	6 (2)	0,53
Coitus interruptus	15 (2)	8 (3)	0,14
Intrauterine contraception	23 (3)	3 (1)	0,07
Natural family planning	15(2)	2 (1)	0,18
Emergency contraception	14(2)	5 (2)	1
Other	15(2)	11 (4)	0,11

P-value calculated by Fischer´s exact test,

*p-value of 0,05 or less. Women stated all methods they were aware of. Women who stated “intra uterine contraception” could not specify which kind of intrauterine contraception they were aware of.

There were considerable differences in the awareness of different methods depending on age group. Women below 30 years of age spontaneously stated that they were aware of the existence of the vaginal ring (78/429, 18.2%), which is a comparatively new method, significantly more often than women over 30 years of age (37/572, 6.5%, p<0,001). Women above 30 years were significantly more aware of the diaphragm which is in practice currently only very rarely used in Sweden (p<0.001).

**Fig 3:**
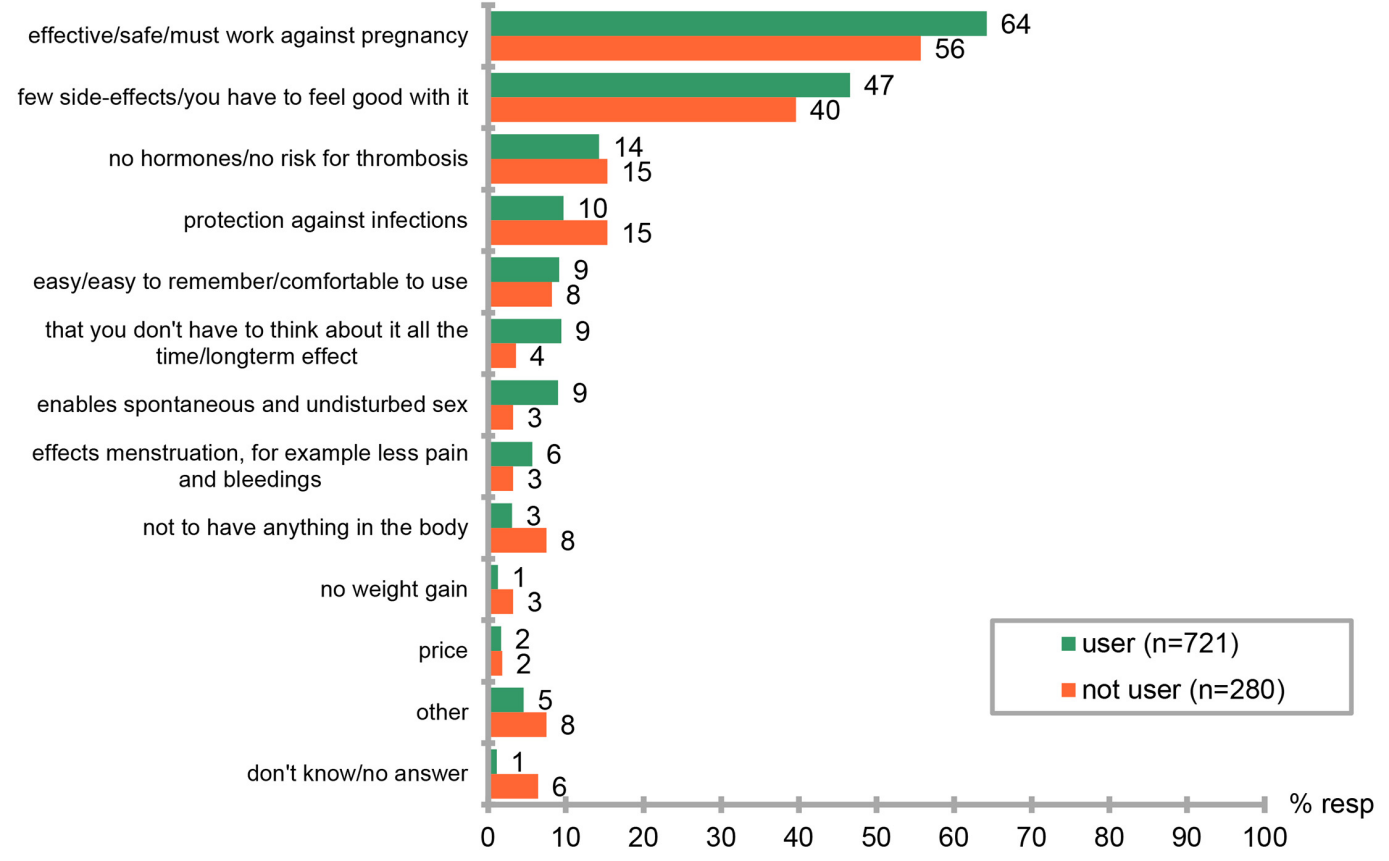
Important characteristics when choosing a contraceptive method.

**Fig 4:**
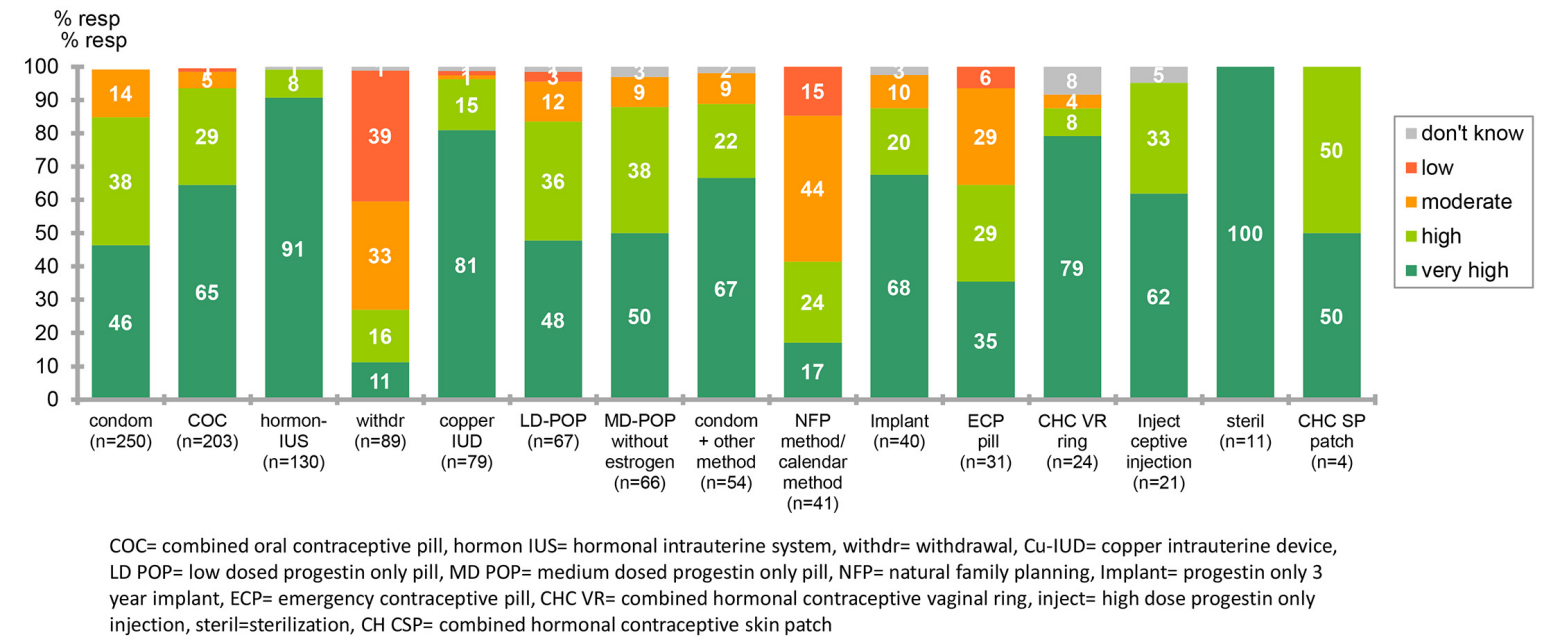
Perception of effectiveness of contraceptive method currently in use (n = 721).

Users and non-users alike stated that one of the most important characteristic of a contraceptive method is its effectiveness. However, significantly more women using contraceptives (463/721, 64.2%) stated that this was important compared to non users (146/280, 52.1%, p = 0.0138, Fishers´s exact test).The importance of different characteristics of contraceptive methods are given in Fig 3. Women were asked to state the perceived effectiveness of the contraception that they had used during the 12 months prior to the survey. The perceived effectiveness of the different methods is shown in Fig 4.

Of the women who were using or had used the contraceptive pill at some point (n = 849) a majority of women had experienced side effects. A majority of women also admitted to forgetting to take one or several pills. A total of 13% (110/849) stated that this happens/happened often. The details on perceived problems with pill usage are outlined in S1 Diagram. Use of the male condom was also associated with perceived problems which are outlined in S2 Diagram.

In total 64.7% (648/1001) of women would find it positive to be without menstrual bleeding for a longer period of time. Out of the 1001 women in the survey 30.9% (309/1001) did not want to be without menstrual bleeding and 4.4% (44/1001) were undecided. There was no difference in preference depending on the age of the women (results not shown). The vast majority of women (891/1001, 89%) had used a contraceptive pill at some point. Intake of the combined hormonal contraceptive pill without the pill free/placebo break in order not to have the monthly bleeding was routinely done by 100/891 (11.2%) women, done once by 62/891 (7%) women, and sometimes by 215/891 (24.1%) women. A total of 513/891 (57.6%) women had never skipped a pill free/placebo break.

When asked actively about use of emergency contraception only six women (6/1001, 0.6%) stated that they were not aware of this method. Of the 1001 women, a total of 781 women (781/1001, 78%) had never used emergency contraception, 155 women (155/1001, 15.5%) had used it once, 39 women (39/1001, 3.9%) had used it twice, and 23 women (23/1001, 2.3%) had used it between 3 and 9 times. Regular use of emergency contraception was stated by 3 women (3/1001, 0.3%).

The majority of women (781/1001, 78%) stated that they had never had an unintended pregnancy whereas 220 (22%) women had experienced at least one unintended pregnancy. There were a total of 326 unintended pregnancies. Outcomes of unintended pregnancies are shown in S3 Diagram. Out of the 326 unintended pregnancies 148 pregnancies resulted in childbirth. A total of 130 pregnancies resulted in abortion, 42 in miscarriage and 4 pregnancies were ectopic.

In total 185/220 (84%) women had experienced an unintended pregnancy without using contraception and a total of 185/326 (56,7%) unintended pregnancies arose when the women did not use contraception. For the first unintended pregnancy arising without contraception the number of women was 114 (114/220, 51.8%). The numbers for the second, third, fourth, fifth and sixth unintended pregnancy were 44 (44/65, 67.7%), 19 (19/26, 73.1%), 3/7, 3/4, and 2/3 respectively. For pregnancies where contraceptive use failed the methods used for these pregnancies are shown in Table 3.

**Table 3:** Contraceptive method used at time of conception of experienced unintended pregnancies.

Contraceptive method	UP1(n = 106)(%)	UP2 (n = 21)(%)	UP3 (n = 7)(%)	UP4 (n = 4)(%)	UP5 (n = 1)(%)	UP6(n = 3)(%)	Total(n = 326)(%)
Intra uterine hormonal system	3(2,8)						3(9,2)
Copper intrauterine device	2(1,8)	2(9,5)	1(14,3)				5(1,5)
Injection	1(0,8)						1(0,3)
Progestin only pill	15(14,2)	1(4,8)	1(14,3)	1(14,7)			14(4,3)
Combined oral contraception	36(34)	13(61,9)	2(28,6)				51(15,6)
Male condom	20(18,9)	1(4,8)					21(6,4)
Coitus interruptus	10(9,6)	2(9,5)	1(14,3)				13(4)
Natural family planning	14(13,2)	1(4,8)	1(14,3)				16(4,9)
Emergency contraceptive pill	1(0,8)						1(0,3)
Combined hormonal contraceptive ring	1(0,8)						1(0,3)
Personal fertility monitor	1(0,8)		1(14,3)				2(0,6)
Do not remember	2(1,8)	1(4,8)		3(42,9)	1(25)	1(33,3)	8(2,5)

UP = Unintended pregnancy.

Considering all unintended pregnancies, 69 women (69/220, 31.3%) women had experienced an unintended pregnancy when they were currently using oral contraception and a total of 69/326 (21,2%) of unintended pregnancies arose while using contraceptive pills. For 21/220 women (9.5%) a male condom was used at the time of conception of the unintended pregnancy and the condom was thereby used at the time when 21/326 (6,4%) unintended pregnancies resulted. Less effective methods such as coitus interruptus and fertility awareness based methods were used at the time when 29/220 (13.2%) of women had an unintended pregnancy and were used when 29/326 (8,9%) unintended pregnancies resulted.

We define “unmet need for contraception” in Sweden as non contraceptive users who are not trying to get pregnant, are not infertile or who do not exclusively have same sex partners. Among the 268 non contraceptive users in our sample, 65 were trying to conceive, 100 were “infertile or partner infertile or sterilized”, 8 were menopaused, and 6 had exclusively same sex partners, leaving a total of 89 non users with a need for contraception. The unmet need for contraception in Sweden is thus 8.9% (89/1001 women). In addition 303/721 (42%) used less effective methods (other methods than hormonal, intrauterine or permanent contraception). Women in the age group 21–30, who have the highest number of unwanted pregnancies, had an unmet need for contraception of 13.4% (35/262).

## Discussion

This nationwide survey shows that a high proportion of women in Sweden have an unmet need for contraception (89/1001, 8.9%). The unmet need for contraception is highest (35/262, 13.4%) in the age group with the highest number of unwanted pregnancies. A total of 781 women (78%) had never experienced an unintended pregnancy whereas 220 women (22%) had had at least one unintended pregnancy. This also confirms the high proportion of women in Sweden who experience unintended pregnancies.

A strength of this survey is the large sample size of 1001 women. There are also few missing answers for all questions in the survey which contributes to high data quality. The major limitation of the survey is that many women could not be contacted and therefore were replaced by other women in the same age category. As women had no information on why they were being called, a selection bias based on a wish to avoid the subject of the survey is highly unlikely in women not being reached. However, contraceptive behavior is not only influenced by the socio-demographic situation, but by type of partnerships and lifestyles that may also be related to not being reached by telephone. A selection bias can therefore not be excluded. A number of women denied participation after having had the survey explained to them. However, the majority of these women denied participation due to a principle of not answering surveys and not the subject of the survey. There was a slight but lower proportion of immigrant women in our sample. It is known that immigrant women often have lower contraceptive use. Therefore, this under representation of immigrant women is unlikely to lead to underestimation of contraceptive use.

There were considerable differences in the awareness of different methods depending on age group. Women of all age groups should be continuously informed of new contraceptive methods so that they can actively choose from all available methods. It may be that women are not using the contraceptive methods which best suits them, but rather the method which they have once received and since then have passively continued using.

The unmet need for contraception in Sweden is as high as 8.9%. This can be compared to the United States (10.7%) [11]) and France (<3%)[12]. The unmet need of contraception in Sweden is difficult to explain as contraception is easy to access in maternal and youth health care centers and is often subsidized for youth. In addition a large proportion of Swedish women (42% of contraceptive users) use methods with a lower effectiveness. This is a considerably higher proportion than in France where only 15% of women use less effective methods[13]. Although the USA has a higher unmet need for contraception the proportion of women using an effective method is higher[14]. A total of 24.3% of women in this survey used LARC. In Sweden as well as other countries the less effective methods are over represented in the methods of contraception used at the time of unintended pregnancy due to their low effectiveness. The use of less effective methods and the proportion of women with an unmet need for contraception in Sweden may well account for the differences in abortion rates between France and Sweden.

Users and non-users alike stated that one of the most important characteristic of a contraceptive method is its effectiveness. However, the estimated effectiveness of the method that women themselves had used in the last 12 months was grossly overrated in the survey. Women had very high confidence in the effectiveness of the male condom and combined oral hormonal contraception. They also underestimated the efficacy of all LARC methods. Similar results on under estimation of effectiveness of LARC methods have been shown in a study from the United States[2]. Overestimation of the effectiveness of the method the women are using may well contribute to why both these countries have a high proportion of unwanted pregnancies and abortion. Information on effectiveness of all contraceptive methods in typical use should be stressed in contraceptive counseling.

It has been shown that contraceptive use at the first intercourse is approximately 75% among Swedish female teenagers. However, only 15% of Swedish teenage women used oral contraception at the time of their first intercourse[15]. It is striking that 66% (31/47) of women in the age group 16–20 years stated that they did not use contraception because they are not sexually active. The mean age of sexual debut in Sweden is slightly higher than 16 years. Thus, although most of the women stating “not being sexually active” as a reason for not using contraception they will have had sex. Yet, they choose not to use contraception. This is well in line with clinical experience where counseling staff in contraception often experience that women do not use contraception in between partners/relationships. It has been shown that 77% of teenage Swedish women used contraception at their last intercourse[15]. Thus, the proportion of teenage women not using contraception at their last intercourse is higher in Sweden (23%) than in eg Finland (17%)[16]. Increasing use of effective contraception prior to having intercourse and in between partners is a way forward to reduce rates of unwanted pregnancies and can be achieved by closer cooperation between schools and youth clinics. This has also been a recommendation put forward by the last national investigation into the high abortion rates in Sweden[17]. LARC use is also lowest in the age groups experiencing the highest number of unwanted pregnancies resulting in abortion. Increasing the proportion of young women using LARC could therefore lower rates of unintended pregnancies, unwanted pregnancies and abortion. The use of LARC methods among young women could be more firmly recommended in guidelines of contraceptive use. Increased LARC use would most probably increase contraceptive use in between partners/relationships and increase contraceptive use at last intercourse in this age group.

In the question concerning unintended pregnancies user dependent methods such as oral contraceptive pills, male condoms and fertility awareness based methods were the most common methods used at the time of conception. Only 9/326 (2.8%) of unintended pregnancies were experienced while using LARC. There have been several government initiated investigations into the relatively high abortion rate in Sweden[17–21]. Several of these investigations have concluded that the other Nordic countries which have been successful in lowering the number of abortions have had national programs for sexual and reproductive health with the object of prevention of unintended pregnancies and recommended that this should be the case also in Sweden. However, so far these recommendations have been ignored

Side effects are also a reason for not using contraception and this reason for not using contraception is most common in the youngest age group. This confirms that young women are more likely to quit using a contraceptive method due to perceived side effects. Most women start using contraception during a period in life when they mature into adult women. This is a difficult period in life for most women. Counselors in contraception should therefore balance the perceived side effects to the current situation in the young woman´s life in order to find a contraceptive method with minimal perceived side effects.

This survey of contraceptive use and attitudes towards contraception shows that many Swedish women at risk of unwanted pregnancy do not use contraception in spite of easy access and a system with subsidies for young women. Women believe that contraceptive effectiveness is the most important quality of contraception but knowledge about contraceptive effectiveness is lacking and a high proportion of women using contraception use less effective methods. A large proportion of women have experienced at least one unintended pregnancy. Increasing awareness of contraceptive effectiveness and promoting use of all contraceptive methods and especially LARCs in guidelines is a possible way forward in the effort to reduce the rates of unintended pregnancy, unwanted pregnancy and abortion.

## Supporting Information

S1 Diagram Problems experienced during use of the contraceptive pill. (XLSX)

S2 Diagram Problems experienced during use of the the male condom. (XLSX)

S3 Diagram Outcomes of unintended pregnancies. (XLSX)

S1 Document Supplemental Information. (DOCX)

S1 Database Original database from TNS SIFO. (XLSX)
