# Supplementary material for: Use of Contraception and Attitudes towards Contraceptive Use in Swedish Women - A Nationwide Survey
Source: PLoS One. 2015 May 20;10(5):e0125990. doi: 10.1371/journal.pone.0125990 (PMC4439158; doi:10.1371/journal.pone.0125990)
Supplement: S1 Document — (DOCX) [file pone.0125990.s004.docx]

Supplemental information 1

Database PAR KONSUMENT TM

The database PAR KONSUMENT TM is a market leading database owned by a commercial company. It derives information from telephone operators in Sweden with new data being supplied on a daily basis. Alla present data is also updated daily. The data transferred to the database from the operators contains all telephone numbers used by individuals in Sweden who have a name attached to the number. In addition the personal identification number of these individuals is supplied to the database. The telephone numbers are bothed fixed and mobile.

The personal identification number is subsequently used to collect information about these individuals from the national Tax authority in Sweden. Also, the adresses of the fixed line telephone number is matched to all individuals who are registered as residing at that adress using the census information in the registers of the Tax Authority.

Sweden has a complete coverage of all individuals residing in Sweden. The personal identification number is used to identlify all individuals residing in Sweden irrespective of natioanality. In Sweden the national personal identification number is used in all contacts with the goverment and also with commercial companies as a means of identifying indivuals.

PAR KONSUMENT TM thus covers 6 900 000 Swedes of ages 16 and above and is considered the most complete database of individuals with phone numbers in Swedem

There are several limitations to the database. Younger individuals often buy cash phone cards with no personal identification number attached to the phone number. They also more often have phones in the names of their parents. Immigrants have fewer phone numbers per person (personal communication PAR KONSUMENT). Also, the register is not allowed to give out phone numbers to individuals who have denied access to their phone number. Denying access is more common for younger individuals and differs in different regions in Sweden, being more common in big cities (personal communication PAR KONSUMENT).

The company that ownes the database tries to compensate for the shortcomings of the database using possible corrections in the sampling in order to produce a representative sample. Such compensations are recommendations to increase the number of young individuals by a factor of 1.2 (personal communication PAR KONSUMENT).

The distribution of the demographical characteristics of individuals in the offered sample is always compared to the expected distribution supplied by the Tax Authority. The demographical characteristics that are compared are the ones present in the Tax Authority register such as age and areas where people. Therefore, these are the characteristics that can be corrected for. The Tax Authority does not offer statistics on current income or ethnic background to commercial companies when matched to personal identification numbers . Therefore, it is impossible to correct for these characteristics. Thus, the bias which remains cannot be corrected for as long as the study is performed as a telephone survey.
